# Use of proton pump inhibitors and macrolide antibiotics and risk of acute kidney injury: a self-controlled case series study

**DOI:** 10.1186/s12882-022-03008-x

**Published:** 2022-11-30

**Authors:** Keiko Ikuta, Shunsaku Nakagawa, Chinami Yamawaki, Kotaro Itohara, Daiki Hira, Satoshi Imai, Atsushi Yonezawa, Takayuki Nakagawa, Minoru Sakuragi, Noriaki Sato, Eiichiro Uchino, Motoko Yanagita, Tomohiro Terada

**Affiliations:** 1 Department of Clinical Pharmacology and Therapeutics, Kyoto University Hospital, Kyoto, Japan; 2 Graduate School of Faculty of Pharmaceutical Science, Kyoto University, Kyoto, Japan; 3 Department of Nephrology, Graduate School of Medicine, Kyoto University, Kyoto, Japan; 4 Department of Biomedical Data Intelligence, Graduate School of Medicine, Kyoto University, Kyoto, Japan; 5 Institute for the Advanced Study of Human Biology (WPI-ASHBi), Kyoto University, Kyoto, Japan

**Keywords:** Proton pump inhibitor, Macrolide antibiotics, Acute kidney injury, Self-controlled case series study

## Abstract

**Background:**

Proton pump inhibitors (PPIs) are widely used for the treatment of gastrointestinal disorders such as peptic ulcer disease and dyspepsia. However, several studies have suggested that PPI use increases the risk of acute kidney injury (AKI). PPIs are often concomitantly used with antibiotics, such as macrolides and penicillins for *Helicobacter pylori* eradication. Although macrolide antibiotics are considered to have relatively low nephrotoxicity, they are well known to increase the risk of AKI due to drug-drug interactions. In this study, we aimed to investigate the association between PPI use and the development of AKI. We also evaluated the effect of concomitant use of PPIs and macrolide antibiotics on the risk of AKI.

**Methods:**

This self-controlled case series study was conducted using electronic medical records at Kyoto University Hospital. We identified patients who were prescribed at least one PPI and macrolide antibiotic between January 2014 and December 2019 and underwent blood examinations at least once a year. An adjusted incident rate ratio (aIRR) of AKI with PPI use or concomitant use macrolide antibiotics with PPIs was estimated using a conditional Poisson regression model controlled for the estimated glomerular filtration rate at the beginning of observation and use of potentially nephrotoxic antibiotics.

**Results:**

Of the 3,685 individuals who received PPIs and macrolide antibiotics, 766 patients with episodes of stage 1 or higher AKI were identified. Any stage of AKI was associated with PPI use (aIRR, 1.80 (95% confidence interval (CI) 1.60 to 2.04)). Stage 2 or higher AKI was observed in 279 cases, with an estimated aIRR of 2.01 (95% CI 1.57 to 2.58, for PPI use). For the period of concomitant use of macrolide antibiotics with PPIs compared with the period of PPIs alone, an aIRR of stage 1 or higher AKI was estimated as 0.82 (95% CI 0.60 to 1.13).

**Conclusions:**

Our findings added epidemiological information for the association between PPI use and an increased risk of stage 1 or higher AKI. However, we did not detect an association between the concomitant use of macrolide antibiotics and an increased risk of AKI in PPI users.

**Supplementary Information:**

The online version contains supplementary material available at 10.1186/s12882-022-03008-x.

## Background

Proton pump inhibitors (PPIs.) are widely used for the treatment of gastrointestinal disorders such as peptic ulcer disease and dyspepsia. Although PPIs have been recognized to show high efficacy , there are growing safety concerns about PPI causing a potential risk of acute kidney injury (AKI), acute interstitial nephritis (AIN) and chronic kidney disease [1–12]. The effect of PPI use on the development of AKI has been detected in several large observational studies [1–6, 12]. However, those studies defined AKI based on the medical records with ICD-9 or ICD-10 [1–6, 12]. Because AKI is commonly under-recorded in hospital data [13], the absolute risk of AKI in the target population may be underestimated in those studies. Other studies have used laboratory data to detect AKI in PPI users [7–11]. However, these studies have the following limitations. First, they identified H_2_-receptor antagonist users as active comparator [7–10]. With this design, the effect of PPIs could be significantly affected by confounding by indication. Second, it was not investigated whether AKI occurred during the exposure period to PPIs [11]. In addition, no studies have classified the stage of AKI. Therefore, further studies are required to validate whether PPI use is associated with the risk of any stage of AKI or more severe AKI.

PPIs play a pivotal role in the standard of care for *Helicobacter pylori* eradication and are often concomitantly used with antibiotics, such as macrolides and penicillins. Although macrolide antibiotics are considered to have relatively low nephrotoxicity [14], they are well known to increase the risk of AKI due to drug-drug interactions. For example, the concomitant use of macrolide antibiotics with calcium channel blockers [15] or HMG-CoA reductase inhibitors (statins) [16] cause a decrease in the clearance of these drugs, and a resultant increase in circulating drug, thereby increasing the risk of drug-induced AKI. For PPIs, previous studies have shown that clarithromycin, a macrolide antibiotic, decreases the clearance of omeprazole [17], lansoprazole [18], and esomeprazole [19]. However, it is unclear whether the risk of AKI is affected by the interactions between PPIs and macrolide antibiotics. Therefore, further studies are required to investigate the effect of macrolide antibiotic use on the risk of AKI in PPI users.

Based on the above backgrounds, we investigated the association between PPI use and the development of AKI using a self-controlled case series (SCCS) design [20–22]. This design allowed us to assess the effects of PPI use on renal function while minimizing chance of potential and unmeasured confounding factors. We also evaluated how the relative risk of AKI in PPI users was associated with concomitant use of macrolide antibiotics.

## Methods

### Study population

We identified all patients who were prescribed at least one PPI and macrolide antibiotic between January 2014 and December 2019 , by searching electronic medical records at the Kyoto University Hospital. The records cover information of all patients who had ever consulted doctors in the hospital, such as age, sex, laboratory data, and prescription data, but data recorded in other hospitals were not included. Table S1 shows the definitions of the study drugs (PPIs and macrolide antibiotics). We then excluded patients who were younger than 18 years of age when PPIs were prescribed or when serum creatinine levels were measured. In addition, we excluded patients who did not undergo blood examinations at least once a year. We also excluded patients whose estimated glomerular filtration rate (eGFR) value at baseline was less than 8 mL/min/1.73m^2^ because they were considered to have end-stage renal disease [22]. We also excluded patients who had a record of macrolide antibiotic use within 14 days before the initial PPI prescription and patients in whom the periods of macrolide antibiotics exposure did not overlap with the initial PPI exposure period. This study was approved by the Ethics Committee of Kyoto University Graduate School and Faculty of Medicine on April 26, 2021 (R2957).

### Study design

We used a SCCS study, which compares within individuals rather than between individuals [20, 21]. Only patients who experienced both AKI and exposure of the study drugs during the observational periods were included. The advantage of this methods is that it cancels out for confounders that do not vary over time, such as comorbidity and chronic medication use.

### Definition of AKI

The criteria for AKI used in this study were based on those used in previous studies [23, 24], which roughly corresponded to the Kidney Disease: Improving Global Outcomes (KDIGO) creatinine criteria [25]. We defined the following criteria for stage 1 or higher AKI. Criterion 1 was defined as an increased serum creatinine level at least 1.5 times higher than the median of all creatinine levels 8-365 days before. Criterion 2 was defined as serum creatinine level increased to at least 1.5 times that of the reference value within 7 days. Criterion 3 was defined as an increase greater than or equal to 0.3 mg/dL within 2 days. We also defined stage 2 or higher AKI as follows: serum creatinine elevation greater than or equal to twice the reference level. Recovery from AKI was defined as the return of serum creatinine to within 25% of the reference value at any time during the observational period [26]. In addition, when a patient recovered from AKI and developed AKI again, each event was considered independent. A second or subsequent incidence of AKI was considered a recurrence if it occurred more than 7 days after the previous AKI incidence [22]. We assumed that the incidence of AKI was influenced by preceding exposure. Therefore, when an AKI episode was detected on the first day of exposure period, we characterized it as left-censored.

### Definition of exposure

**Fig. 1:**
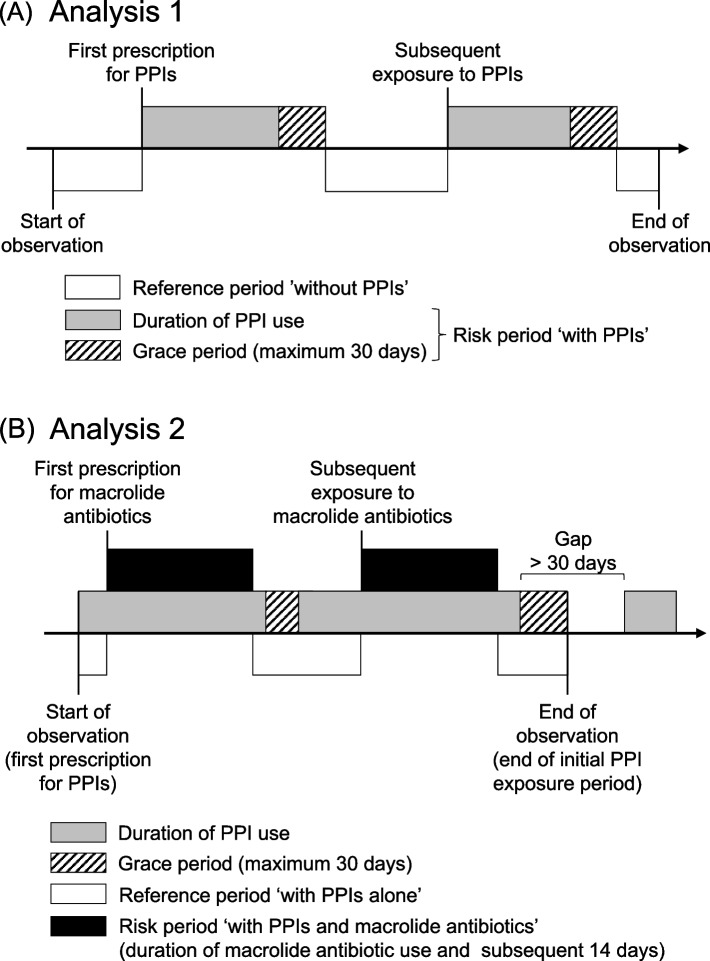
Study design and definition of exposure. **A** In analysis 1, we aimed to investigate the association between proton pump inhibitor (PPI) use and the development of acute kidney injury (AKI). The start of the observation was defined as the earliest date of the first serum creatinine measurement or PPI prescription. The end of observation was defined as the latest data of the last serum creatinine measurement, 30 days after the end of last administration of a PPI, or date of death. PPI exposure period (risk period) was defined as the period from the start of PPI administration to 30 days after the end of administration. **B** In analysis 2, we evaluated the effect of concomitant use of macrolide antibiotics on the risk of AKI only during the initial PPI exposure period. The exposure period of macrolide antibiotics (risk period) was defined as the period from the start of macrolide antibiotics administration to 14 days after the end of its administration

Fig. 1 describes the observation and exposure periods. First, we aimed to investigate the association between PPI use and the development of AKI (analysis 1). The start of the observation period was defined as the earliest date of the first serum creatinine measurement or PPI prescription. The end of observation was defined as the latest date of the last serum creatinine measurement, 30 days after the end of last administration of a PPI, or date of death. We assumed that drug administration began on the day of prescription and continued for the number of prescription days recorded in the database. PPI exposure period was defined as the period from the start of PPI administration to 30 days after the end of administration. The SCCS study is a type of cohort study in which relative risk is based on a within-person comparison between exposed and unexposed to a drug [20, 21]. Therefore, the observation period was divided into the PPI exposure period (risk period ‘with PPIs’) and the unexposed period (reference period ‘without PPIs’).

Then, we evaluated the effect of concomitant use of PPIs and macrolide antibiotics on the risk of AKI (analysis 2). The exposure period of macrolide antibiotics was defined as the period from the start of macrolide antibiotics administration to 14 days after the end of its administration. Based on the previous study examining the effect of drug combinations on the risk of AKI using a SCCS study [27], we assessed the effect of macrolide antibiotics on the incidence of AKI only during the initial PPI exposure period. We constructed continuous period of PPI exposure by allowing for a 30-day gap between consecutive prescriptions (≤ 30 days between the end of one prescription and start of the next). The initial PPI exposure period was divided into periods of macrolide antibiotic exposure (risk period ‘with PPIs and macrolide antibiotics’) and other (reference period ‘with PPIs alone’).

### Statistical analysis

We described the baseline characteristics of patients using counts (proportions) for categorical variables and means (standard deviation [SD]) or median (interquartile ranges [IQR]) for continuous variables. For the SCCS study, we calculated the IRRs and 95% CIs using conditional Poisson regression with a generalized linear model. Since eGFR and administration of nephrotoxic antibiotics affect the risk of AKI [12, 28], the IRRs and 95% CIs were adjusted for eGFR at the beginning of observation (baseline eGFR) and use of potentially nephrotoxic antibiotics (Table S2). The relationship between the baseline eGFR and the risk of AKI was considered linear. We assumed that potentially nephrotoxic antibiotic use was defined as a time-varying confounder that would correlate to PPI or macrolide antibiotic use and affect the risk of AKI development [12]. Other potentially confounders, such as comorbidity and the administration of other nephrotoxic drugs, were not included in the analysis. All analyses used R version 4.4.1 software and the ‘gnm’ package [22].

### Sensitivity analysis

A study using the SCCS design require the following assumptions [21]. First, event rates should be constant within each defined period. Second, events should be independently recurrent. Third, the occurrence of an event should not affect subsequent exposures. This assumption also means that the event itself should not determine the timing of the end of the observation period. However, severe AKI can cause death or dialysis dependence [29, 30], which results in the end of the observation period and the violation of the assumptions of the SCCS design. Therefore, we performed sensitivity analyses to assess the robustness of the results. Analysis 1 was repeated excluding cases who died during the observation period. Similarly, analysis 2 was repeated excluding cases who died during the initial PPI exposure period.

## Results

### Baseline characteristics

**Fig. 2:**
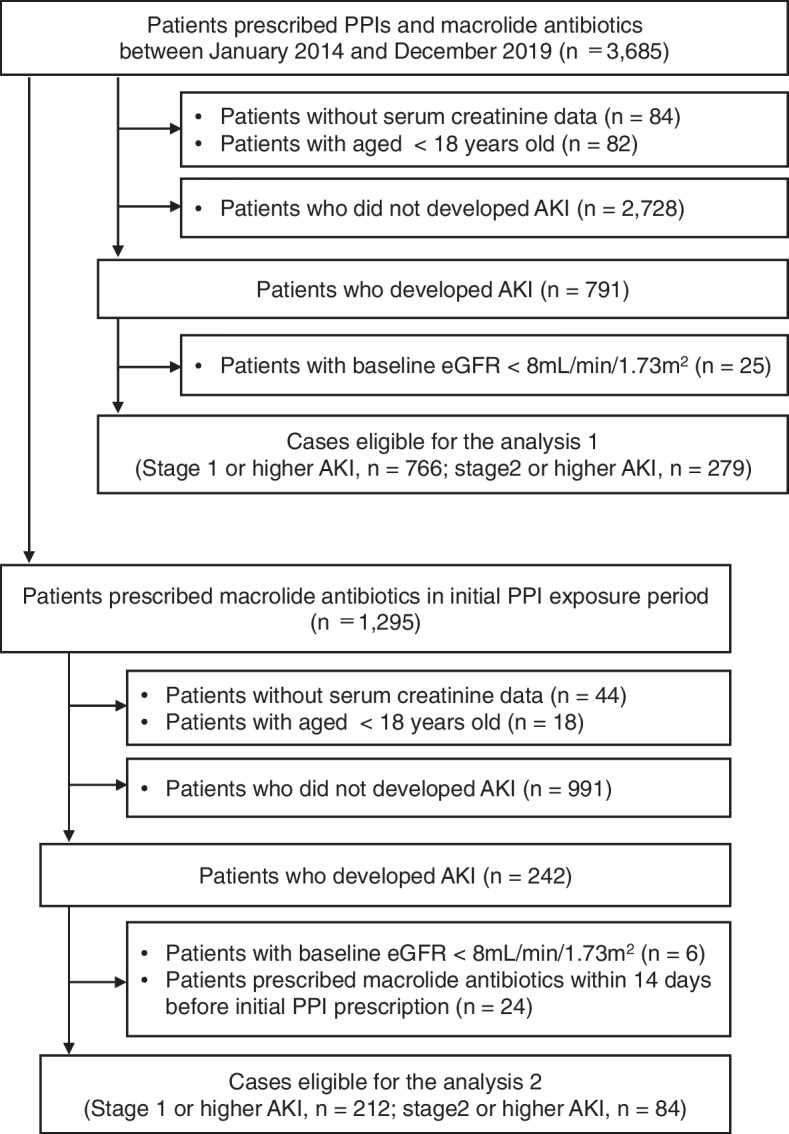
Identification of cases eligible for the analysis. eGFR, estimated glomerular filtration rate

Of the 3,685 individuals who received PPIs and macrolide antibiotics between January 2014 and December 2019, 766 cases with stage 1 or higher AKI were identified during the observational period (Fig. 2). The clinical characteristics of the patients are summarized in Table 1. The average age at the beginning of observation was 62.9 years (SD, 16.3 years) and 43.0% were women. The median observation period was1,624 days (IQR, 961 to 2,111 days) and the median duration of exposure to PPIs was 485 days (IQR, 160 to 1,346 days). The percentages of patients who had baseline eGFR more than 60, 30–60, and less than 30 mL/min/1.73m^2^ were 65.5, 26.1 and 8.4%, respectively.

**Table 1:** Baseline characteristics of cases with developed stage 1 or higher AKI

Characteristics	Analysis 1 (*n* = 766)	Analysis 2 (*n* = 212)
Age (years), mean (SD)	62.9 (16.3)	65.4 (17.0)
Female, n (%)	329 (43.0)	100 (47.1)
Baseline eGFR (mL/min/1.73 m^2^), n (%)		
More than 60	502 (65.5)	130 (61.3)
30–60	200 (26.1)	59 (27.4)
Less than 30	64 (8.4)	23 (10.8)
Days of observation, median (IQR)	1,624 (961–2,111)	945 (343–1,657)

*eGFR* estimated glomerular filtration rate

Of the 766 cases with stage 1 or higher AKI, 212 individuals were prescribed macrolide antibiotics during the initial PPI exposure (Fig. 2). The average age at the beginning of initial PPI prescription was 65.4 years (SD, 17.0 years) and 47.1% were women. The median length of initial PPI exposure was 945 days (IQR, 343 to 1,657 days), including 21 days (IQR, 17 to 45 days) of exposure to macrolide antibiotics. The percentages of patients who had baseline eGFR more than 60, 30–60, and less than 30 mL/min/1.73m^2^ were 61.3, 27.4 and 10.8%, respectively (Table 1).

### Analysis 1: association between PPI use and IRRs of AKI

During the observation period, 766 individuals who developed a total of 1,317 episodes with stage 1 or higher AKI (Table 2). Of these, 373 occurred without exposure to PPIs and 944 occurred with exposure to PPIs. For the period with PPIs compared to that without PPIs, IRR and aIRR were estimated as 2.16 (95% CI, 1.91 to 2.43) and 1.80 (95% CI, 1.60 to 2.04), respectively.

**Table 2:** Association between PPI use and incident rate ratio of AKI

	Number of episodes	Person-day	IRR (95% CI)	Adjusted IRR* (95% CI)
Stage 1 or higher AKI				
Without PPIs	373	518,234	Reference	Reference
With PPIs	944	607,707	2.16 (1.91–2.43)	1.80 (1.60–2.04)
Stage 2 or higher AKI				
Without PPIs	87	189,032	Reference	Reference
With PPIs	240	211,611	2.46 (1.93–3.15)	2.01 (1.57–2.58)

*IRR* incident rate ratio, *AKI* acute kidney injury, *CI* confidence interval, *PPI* proton pump inhibitor. *Adjusted for the use of potentially nephrotoxic antibiotics and baseline eGFR

We then performed the analysis by including only cases with stage 2 or higher AKI (Table 2). During the observation period, 279 individuals who developed a total of 327 episodes with stage 2 or higher AKI. The IRR and aIRR for the period with PPIs compared to that without PPIs were estimated as 2.46 (95% CI, 1.93 to 3.15) and 2.01 (95% CI, 1.57 to 2.58), respectively. In the sensitivity analysis, the significantly increased IRRs of AKI were still observed by PPI use (Table S3).

### Analysis 2: effect of concomitant use of PPIs and macrolide antibiotics on IRRs of AKI

The risk of AKI associated with the concomitant use of PPIs and macrolide antibiotics was evaluated because macrolide antibiotics are known to alter the pharmacokinetics of PPIs [17–19]. During the initial PPI exposure period, 212 individuals who developed a total of 385 episodes with stage 1 or higher AKI. Of these, 49 and 336 episodes occurred with and without macrolide antibiotic exposure, respectively. For the period with PPIs and macrolide antibiotics compared to that with PPIs alone, the IRR and aIRR were estimated as 1.26 (95% CI, 0.94 to 1.71) and 0.82 (95% CI, 0.60 to 1.13), respectively (Table 3).

**Table 3:** Association between exposure to macrolide antibiotics and IRR of AKI in PPI users

	Number of episodes	Person-day	IRR (95% CI)	Adjusted IRR* (95% CI)
Stage 1 or higher AKI				
With PPIs alone	336	196,105	Reference	Reference
With PPIs and macrolide antibiotics	49	22,626	1.26 (0.94–1.71)	0.82 (0.60–1.13)
Stage 2 or higher AKI				
With PPIs alone	89	69,484	Reference	Reference
With PPIs and macrolide antibiotics	13	10,006	1.01 (0.57–1.82)	0.73 (0.40–1.34)

*Adjusted for the use of potentially nephrotoxic antibiotics and baseline eGFR

We also identified 84 individuals who developed a total of 102 episodes with stage 2 or higher AKI during the initial PPI exposure period. For the period with PPIs and macrolide antibiotics compared to that with PPIs alone, the IRR and aIRR were estimated as 1.01 (95% CI, 0.57 to1.82) and 0.73 (95% CI, 0.40 to 1.34), respectively (Table 3). Sensitivity analysis did not detect an association between the combination of PPIs and macrolide antibiotics and increased IRR of AKI (Table S4).

## Discussion

With PPIs being one of the most commonly used classes of drugs, the potential for adverse reactions to PPI use should be clarified. Recently, an association between PPI use and increased risk of AKI has been reported [1, 2, 4–12]. However, these studies have the following shortcomings in their study design: First, the severity of the AKI was not evaluated [1, 2, 4–6, 12]. Second, the effect of confounding by indication was not eliminated [6–10]. Third, temporal proximity between PPI exposure and AKI onset was not considered [11]. Therefore, the main objective of this study was to confirm the association between PPI use and the risk of any stage of AKI or more severe AKI (stage 2 or higher). To our knowledge, this is the first study to use a SCCS study and serum creatinine data to investigated the association between PPI use and the risk of AKI. We also evaluated the effect of the concomitant use of macrolide antibiotics on the risk of AKI in PPI users.

This study confirmed that PPI use is associated with an increased risk of AKI. The direction and significance of the results are consistent with those of previous studies [1, 2, 4–8, 10–12]. However, the severity of AKI has not been evaluated in previous studies. Some studies measured AKI using ICD codes [1, 2, 4, 5, 12], but this method does not provide information on the severity of AKI [13]. Other studies used serum creatinine data to measure the incidence of AKI [7–11] but only determined whether it was stage 1 or higher. On the other hand, we found that more severe AKI (KDIGO stage 2 or 3) was associated with PPI use, which expands on the findings of previous studies. Taken together, these results indicated that PPI use is independent risk factors for the development of AKI. Given this notion, further attention should be paid to the development of AKI during the course of treatment with PPIs.

Macrolide antibiotics have the potential to alter the pharmacokinetics and risks of adverse reactions to commonly used drugs because they are enzyme inhibitors [15, 16]. In previous studies, macrolide antibiotics increased the serum concentration of PPIs [17–19], implying that its concomitant use with macrolide antibiotics may alter the risk of AKI in PPI users. Therefore, we investigated the effects of PPIs and macrolide antibiotics on the risk of AKI. The results of this study suggest that the association between macrolide antibiotic use and AKI risk among PPI users is not significant. Although the pharmacokinetics of PPIs was not evaluated in this study, our results suggest that the exposure levels of PPIs are not related to their nephrotoxicity. This is supported by a previous study showing that the genotype or phenotype of CYP2C19, a PPI-metabolizing enzyme, does not affect the risk of AIN in PPI users [31]. AIN is the most frequently reported pathology in patients with PPI-related AKI [1–3, 32, 33]. These results suggest that indirect mechanisms rather than direct nephrotoxicity may be involved in the development of PPI-related nephropathy. In previous studies, it was suspected that several mechanisms of drug hypersensitivity reactions play a role in the pathogenesis of PPI-related AKI and that these mechanisms overlap [34]. On the other hand, there is growing evidence that the reduction of gastric acidity induced by PPI use leads to changes in the gut microbiome and that PPI-induced dysbiosis is associated with the progression of PPI-related adverse effects [35]. For example, omeprazole has been shown to increase the expression of inflammatory cytokines in liver tissue and increases its vulnerability to hepatic injury, which occurs via changes in the gut microbiota [36]. It should also be investigated whether changes in the gut microbiota and subsequent changes in inflammatory status are related to PPI-associated nephropathy.

This study has several strengths. First, we used the SCCS study which compares risk within individuals at different periods and thus, the results are less influenced by potential confounding between comparison groups than cohort studies or case control studies. In the present study, we only included patients who developed AKI and used PPIs and macrolide antibiotics at least once, which minimized confounding by indication. Second, the IRR was adjusted by the time method using a regression model. Third, we used serum creatinine data to measure the development of AKI with reference to the National Health Service (NHS) England AKI algorithm criteria [23–25]. We also assessed the stage of AKI. A recent study validated the AKI algorithm by comparing a clinical diagnosis by experienced nephrologists with the NHS England criteria [37]. The study indicated that 90.5% of patients with a clinical diagnosis of AKI was detectable using the AKI algorithm of the NHS. Therefore, we assumed that AKI could be detected with a high sensitivity in this study. Fourth, since low renal function is itself a risk factor for AKI, we adjusted the incidence ratio by baseline renal function.

This study has several limitations. First, we only included patients who underwent blood examinations at least once a year in the study hospital and did not filter by the frequency of the examination. The frequency of blood examinations in patients who did not develop AKI was 11.0 ± 14.9 times a year and that of those who did develop AKI (named the ‘cases’) was 27.2 ± 25.8 times a year. It is possible that few patients underwent blood examination at other hospitals. Moreover, the data on the urine volume is missing. Therefore, it is possible that there was a delay in identifying the timing of AKI onset or misclassification of non-cases. Second, we did not consider liver function or systemic inflammation to be time-varying factors. The reason for this setting was to avoid missing data, which could decrease the number of cases and the power of detection. However, the criteria for selection of the study population may have led to decreased sensitivity of AKI detection and modification of the results by unmeasured confounders. For example, rapid changes in renal functions due to primary disease or comorbidities could influence the development of AKI. Third, we did not include comorbidities and administration of nephrotoxic agents other than antibiotics as confounding factors. Fourth, severe AKI could lead to death or dialysis dependence [29, 30] and observation may have been terminated due to the occurrence of severe AKI. However, the results of main analysis were consistent in the sensitivity analysis that excluded cases in which observation was terminated due to death (Tables S3 and S4). Fifth, the timing of drug administration was based on prescription records. Therefore, the actual timing of drug administration may be different. In addition, adherence to medication is unknown. Future studies should include prospective measurements of serum creatinine levels and use databases to accurately determine the duration of drug exposure.

## Conclusion

This SCCS study indicated an association between PPI use and an increased risk of any stage of AKI. Further studies are warranted to clarify when the risk of AKI is highest during PPI use and how to predict the AKI development in PPI users. In contrast , we did not detect an association between the concomitant use of PPIs and macrolide antibiotics and an increased IRR of AKI. Given the present results, the concomitant use of macrolide antibiotics in PPI-users may not affect the risk of AKI.

## Supplementary Information

**Additional file 1.**

## Data Availability

The datasets generated and/or analyzed during the current study are not publicly available due to the policy of the institution but are available from the corresponding author on reasonable request.

## Abbreviations

PPI: Proton Pump Inhibitor
AKI: Acute Kidney Injury
AIN: Acute Interstitial Nephritis
ICD: International Classification of Disease
SCCS: Self-Controlled Case Series
eGFR: estimated Glomerular Filtration Rate
KDIGO: Kidney Disease: Improving Global Outcomes
SD: Standard Deviation
IQR: Interquartile Range
IRR: Incident Rate Ratio
aIRR: adjusted Incident Rate Ratio
95% CI: 95% Confidence Interval
NHS: National Health Service
