# Supplementary material for: Use of proton pump inhibitors and macrolide antibiotics and risk of acute kidney injury: a self-controlled case series study
Source: BMC Nephrol. 2022 Nov 30;23:383. doi: 10.1186/s12882-022-03008-x (PMC9710142; doi:10.1186/s12882-022-03008-x)
Supplement: Supplementary file 1 — Additional file 1. [file 12882_2022_3008_MOESM1_ESM.docx]

| **Table S1** Definition of proton pump inhibitors and macrolide antibiotics | |
| --- | --- |
| Category | Drugs |
| Proton pump inhibitors | esomeprazole, lansoprazole, omeprazole, rabeprazole, and vonoprazan |
| Macrolides | azithromycin, clarithromycin, erythromycin, josamycin, roxithromycin, and spiramycin |

| **Table S2** Potentially nephrotoxic antibiotics | |
| --- | --- |
| Category | Drugs |
| Polymixins | polymixin B |
| Tetracyclines | demeclocycline, doxycycline, tetracycline, and minocycline |
| Penicillins | ampicillin, benzylpenicillin, benzylpenicillin, combination of penicillins |
| Cephalosporins | cefalexin, cefalotin, cefazolin, cefroxadine, cefuroxime, cefaclor, cefotiam, cefmetazole, cefminox, cefotaxime, ceftazidime, ceftriaxone, cefmenoxime, ceftizoxime, cefixime, cefpodoxime, ceftibuten, cefidinir, cefditoren, cefcapene, cefteram, cefepime, cefpirome, cefozopran, and cefoperazone |
| Monobactams | aztreonam |
| Carbapenems | meropenem, doripenem, biapenem, tebipenem pivoxil, imipenem, and pamipenem |
| Aminoglycosides | streptomycin, tobramycin, gentamicin, kanamycin, amikacin, dibekacin, isepamicin, arbekacin, and spectinomycin |
| Fluoroquinolones | ofloxacin, ciprofloxacin, norfloxacin, lomefloxacin, levofloxacin, moxifloxacin, prulifloxacin, pazufloxacin, garenoxacin, sitafloxacin, and tosufloxacin |
| Glycopeptides | Vancomycin and teicoplanin |

**Table S3** Association between PPI use and incident rate ratio of AKI.

|  | | Number of episodes | Person-day | IRR  (95% CI) | Adjusted IRR*  (95% CI) |
| --- | --- | --- | --- | --- | --- |
| Stage 1 or higher AKI | | | | | |
|  | Without PPIs | 253 | 371,832 | Reference | Reference |
|  | With PPIs | 502 | 409,166 | 1.80  (1.55–2.10) | 1.52  (1.30–1.77) |
| Stage 2 or higher AKI | | | | | |
|  | Without PPIs | 61 | 126,372 | Reference | Reference |
|  | With PPIs | 109 | 121,406 | 1.86  (1.40–2.54) | 1.61  (1.17–2.21) |

Cases who died during the observation period were excluded. IRR, incident rate ratio; AKI, acute kidney injury; CI, confidence interval; PPI, proton pump inhibitor. *Adjusted for the use of potentially nephrotoxic antibiotics and baseline eGFR.

**Table S4** Association between exposure to macrolide antibiotics and IRR of AKI in PPI users.

|  | | Number of episodes | Person-day | IRR (95% CI) | Adjusted IRR* (95% CI) |
| --- | --- | --- | --- | --- | --- |
| Stage 1 or higher AKI | | | | | |
|  | With PPIs alone | 220 | 157,111 | Reference | Reference |
|  | With PPIs and macrolide antibiotics | 29 | 16,271 | 1.27  (0.86–1.87) | 1.03  (0.70–1.53) |
| Stage 2 or higher AKI | | | | | |
|  | With PPIs alone | 58 | 47,220 | Reference | Reference |
|  | With PPIs and macrolide antibiotics | 5 | 6,290 | 0.65  (0.26–1.61) | 0.59  (0.24–1.49) |

Cases who died during the initial PPI exposure period were excluded. *Adjusted for the use of potentially nephrotoxic antibiotics and baseline eGFR.
